# Repeated recovery of immature oocytes in a woman with a previous history of empty follicle syndrome

**DOI:** 10.5935/1518-0557.20180068

**Published:** 2019

**Authors:** Tarek K. Al-Hussaini, Ali H. Yosef, Ihab H. EL-Nashar, Omar M. Shaaban

**Affiliations:** 1 Department of Obstetrics and Gynecology, Faculty of Medicine, Assiut University, El-Salama IVF/ICSI Centre, Assiut, Egypt

**Keywords:** empty follicle syndrome, IVF/ICSI, *in-vitro* maturation, immature oocytes

## Abstract

The empty follicle syndrome (EFS) is defined as a failure to aspirate any oocyte (s) from the follicles after ovarian hyperstimulation in preparation for IVF/ICSI. It is a frustrating and vague syndrome; and a controversial one concerning its existence, causes and possible treatment. Recurrent EFS or the recovery of immature oocytes thereafter is a more challenging problem. Delayed injection after leaving the immature oocytes for *in vitro*-maturation (IVM) has been suggested to be a possible option if immature oocytes are retrieved. Here, we present a case of repeated retrieval of a few immature oocytes after a first incidence of EFS. IVM was tried twice for those immature oocytes. Unfortunately, in this case IVM was unsuccessful and the oocytes failed to mature *in vitro*. Assistance is required for future management of these unfortunate couples.

## INTRODUCTION

Empty follicle syndrome (EFS) was first described by Coulam *et al*. (1986) when they failed to aspirate any oocytes from the ovaries of four patients prepared for IVF, and one of them was recurrent in two cycles. It is divided into false EFS, when the level of human chorionic gonadotropins (HCG) on the day of ovum pick up is low and genuine EFS when its level is optimal (Beck-Fruchter *et al*., 2012). In turn, this raises another question about the optimal level of HCG on the day of ovum pick up, which varies in different studies and according to different types of HCG used. A systematic review of empty follicle syndrome has suggested a cutoff level of 40 mIU/mL to differentiate between the two types (Stevenson & Lashen, 2008). Although the estimated prevalence of empty follicle syndrome is up to 7% in the literature, the prevalence of genuine cases was as low as 0.016% in a large cohort including more than 12 thousand IVF patients (Mesen *et al*., 2011). Its exact etiology is unknown and difficult to predict. Different treatment modalities to prevent recurrent EFS were suggested including triggering with GnRH agonists (Lok *et al*., 2003) and a second HCG dose followed by another pick up (Ndukwe *et al*., 1997). Recurrent EFS or retrieval of poor quality immature oocyte after a first incidence of EFS is even more challenging. In this report we present a patient who had EFS in her first trial, followed by repeated retrievals of immature oocytes. IVM was tried for those immature oocytes in her two IVF trials carried out at our unit.

## CASE PRESENTATION

A 32 year-old woman with a ten-year history of primary infertility came to our unit for IVF/ICSI with the diagnosis of bilateral tubal block and uncorrectable tubal damage, without hydrosalpinges, and a normal semen profile for her husband. She had a past history of open myomectomy and two laparoscopies for endometriosis treatment (one of them involved Laparoscopic ovarian drilling). She had a previous IVF attempt at another IVF/ICSI clinic, which ended up as an empty follicle syndrome (EFS) and cycle cancelation. In that trial she was submitted to a standard long agonist protocol with highly purified urinary FSH and triggered with 10.000 IU of hCG. After failure to retrieve any oocytes from one ovary she received an additional dose of 10.000 hCF IU and egg collection was rescheduled 24 hours later. Unfortunately, the second trial ended with no eggs being retrieved.

In the second trial (first at our unit), the basal hormonal profile showed: FSH = 6.5 miu/ml, LH = 4.4 miu/ml and AMH = 4.05 ng/ml. We used a fixed antagonist protocol, using Cetrorelix (Cetrotide, Merck Serono, London, UK) and HMG (Menogon, Ferring, Kiel, Germany) 300 IU for 12 days. Dual trigger was done using 10000 IU HCG (Choriomon, IBSA, Lugano, Suisse) and 0.2 mg triptoreline (Decapetyl, Ferring, Kiel, Germany) and OPU was scheduled 36 days thereafter. On triggering day, her transvaginal ultrasound scan showed seven follicles between 17-20 mm. HCG and Decapeptyl (for triggering) were given by a qualified nurse at the correct time. Before OPU, a blood sample was withdrawn which showed E2 to be 3510 pg/ml and B-HCG = 166.3 miu/ml. It ended with the retrieval of one immature oocyte (Germinal vesicle GV) after thorough repeated flushing and aspiration of all follicles. *In vitro* maturation (IVM) was tried for this immature oocyte. However, it degenerated on the following day.

**Figure 1:**
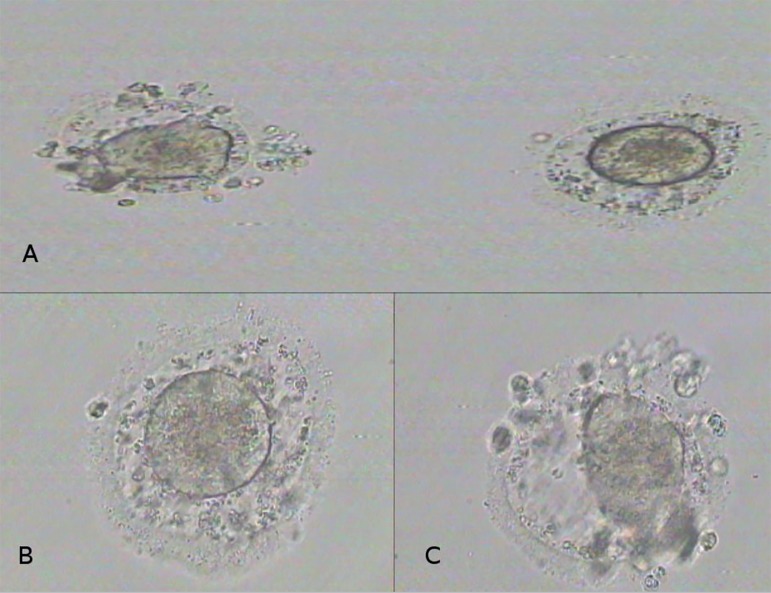
Retrieved immature oocytes. A: Both oocytes together. B, C: Individual oocytes under higher magnification (X 200).

In the third trial (second at our unit), she was thoroughly counseled before starting treatment, when we explained that there was a great possibility of the same thing happening again. She and her husband consented for the third trial. Again, we used a fixed antagonist protocol with Cetrorelix (Cetrotide, Merck Serono, London, UK) and another highly purified HMG (Merional, IBSA, Lugano, Suisse) at a dose of 300 IU per day for 11 days. On the day of triggering, her transvaginal ultrasound scan showed eight follicles between 17-20 mm. Her E2 level before triggering was 1594 pg/ml. Dual trigger was used again, with recombinant hCG (Ovitrelle, Merck, London, UK ), 0.2 mg triptoreline (Decapeptyl, Ferring, Kiel, Germany) and her OPU was carried out 36 hours after triggering. Again, HCG and Decapeptyl (for triggering) were given by a qualified nurse at the correct time. The ß-HCG level was 122.1miu/ml before egg collection and her E2 was 1049 pg/ml. Her egg collection resulted in the retrieval of 2 poor quality immature germinal vesicles despite thorough, repeated flushing and aspiration (Figure 1). Because of her past history of EFS, both her oocyte retrievals at our unit were performed by our most experienced physician. IVM was tried, again, for her two retrieved immature oocytes. Unfortunately, none of them got mature until day five. The patient and her husband gave their informed consent to use their information and photos of their immature eggs for publication as they are quite eager to find a solution for their problem

## DISCUSSION

We are reporting a case of repeated retrieval of immature oocytes in a patient with a previous history of EFS. We have tried *in vitro* maturation for these immature oocytes, which failed to mature further *in vitro*. This patient’s case has multiple noteworthy points of interest. Firstly, she is young and has a good ovarian reserve, as suggested by her basal hormonal profile. Previous case reports suggested a relation between EFS and poor ovarian reserve, and ovarian aging (Beck-Fruchter *et al*., 2012). The exact mechanism for this poor outcome is not known. It might be due to defective folliculogenesis or chromosomal abnormalities. Secondly, she has a previous history of endometriosis that may suggest a role for endometriosis in the pathogenesis of EFS, folliculogenesis or on the quality of eggs retrieved. The association of minimal endometriosis with EFS was reported in a previous cohort study on women undergoing natural IVF cycles (Omland *et al*., 2001). Thirdly, we adopted a combination of different strategies reported in the literature to minimize the recurrence of EFS. The use of an antagonist protocol may release the growing follicles from the strong suppressive effect of a GnRH agonist. Additionally, dual triggering with HCG and GnRH agonist was used, as suggested in a previous case report (Deepika *et al*., 2015). Furthermore, measuring HCG level on the day of ovum pick up confirmed our diagnosis. This rules out the possibility of drug errors (storage or manufacturer problems) and confirms that it is a true syndrome and not a fictional one. Repeated follicular flushing/aspiration during oocyte retrieval was also used. Lastly, we obtained poor quality immature oocytes in the last two trials. A previous case reported retrieval of four immature oocytes following ovarian stimulation using an agonist protocol and an HCG trigger (Vutyavanich *et al*., 2010). However, they did not try IVM. Another group retrieved five zona free GV oocytes after using an antagonist protocol and a GNRH agonist trigger. They tried ICSI for these oocytes but failed (Duru *et al*., 2007). Rescue IVM was tried in this desperate case. It has been suggested that the use of IVM has better results than injecting immature oocytes. Previous trials were carried out on IVM following immature oocyte retrieval with variable results. However, the immature oocytes retrieved in our case failed to mature further *in vitro*.

## CONCLUSION

Empty follicle syndrome seems to be a reality, even with the use of different stimulation protocols and triggering techniques. Recurrent EFS or recovery of immature oocytes after a history of EFS is even more challenging, very stressful and frustrating not only for the couple but also for the whole IVF team involved. The exact etiology of such a condition is still poorly understood. It is most likely caused by abnormal or dysfunctional folliculogenesis. Management options in such difficult cases are very limited. IVM was tried in our patient but unfortunately without success. Such women should be counselled for the very high risk of recurrence in any subsequent IVF/ICSI trial.

### Author's role

Dr. Tarek Al-Hussaini, Dr. Omar Shabaan designed the report. They followed up the patient and Dr. Tarek Al-Hussaini performed the ovum pick up. Dr. Ihab EL-Nashar performed IVM. Dr Ali Yosef assisted in patient follow up. The four authors participated in writing and final approval of the manuscript.
